# Meet key digital health thought leaders: Sandy Engelhardt, Scientific Program Chair of the ESC’s Digital Summit 2025

**DOI:** 10.1093/ehjdh/ztaf060

**Published:** 2025-05-26

**Authors:** Nico Bruining

**Affiliations:** Department of Cardiology, Thoraxcenter, Erasmus MC Rotterdam, Dr. Molewaterplein 40, 3015 GD, Rotterdam, The Netherlands

Cardiopulse Digital talks to Sandy Engelhardt, computer scientist and Professor of Artificial Intelligence in Cardiovascular Medicine at Heidelberg University Hospital. Sandy is currently the Scientific Program Chair of the ESC’s Digital Summit to be held on 21–22 November, Berlin, Germany.


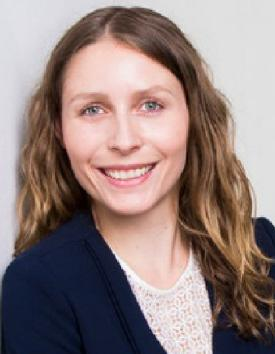


Some information about yourself: I am a computer scientist and Professor of Artificial Intelligence in Cardiovascular Medicine at Heidelberg University Hospital. My primary research goal is to develop AI methods for applications in cardiovascular disease.My dissertation received the BVM Award 2017 for the best PhD thesis, presented by the German Medical Image Processing Community. I serve as the speaker of the Multidimensional AI consortium in the area of HFpEF, funded by the Carl Zeiss Foundation with €5 million.I am very active in several European medical societies. Currently, I hold the Chair for AI in the EACTS Innovation Committee and serve as Subgroup Lead for Education and Outreach within the ESC Digital Cardiology and AI Committee. As Program Chair of the ESC Digital and AI Summit 2025 (https://bit.ly/45gwMoK), I am primarily responsible for shaping the event’s program and bridging the fields of computer science, AI, and cardiovascular research. I also contribute to the ESC Program Committee and the EHRA Digital Medicine and mHealth Committee. Additionally, I support the strategic development of the DZHK AI platform as a DZHK Principal Investigator.

## Your academic and professional journey spans data science and cardiovascular medicine. What inspired you to bridge these two fields, and how has your background shaped your current research focus?

I’ve always been fascinated by the intersection of technology and medicine—particularly how technological advances can be harnessed to support patients and improve healthcare delivery. This interest initially led me to pursue a degree in computer science, but my passion for medical applications remained a consistent thread throughout my studies and academic career.

During my PhD, I had the opportunity to attend numerous open and minimally invasive cardiac surgeries at Heidelberg University Hospital. These experiences deeply inspired me and sparked a lasting interest in cardiovascular medicine. The clinical need for technological support in this field is immense—for both patients and healthcare professionals.

Today, I’m fortunate to lead a technically focused research group embedded within a university hospital setting. My group develops cutting-edge AI methods focussing on the analysis of cardiovascular imaging and diagnostic data (CMR, CT, ultrasound, endoscopy, ECG, etc.) collected at various stages along the treatment pathway. We develop automated risk prediction models or suggest means for more standardization in characterising myocardial deformation —areas where deep learning methods offer promising and highly efficient solutions.

## You have led several innovative projects applying AI to cardiovascular care. Could you share insights from your work on federated learning and how it addresses challenges like data privacy and model generalizability in clinical settings?

Together with my team, we are leading a multicenter Federated Learning initiative (FLOTO), involving eight medical institutions across Germany. The core innovation of this approach lies in its ability to train AI models across distributed sites without requiring patient data to leave the respective hospitals. This preserves data privacy while enabling the secondary use of retrospective clinical data.

We’ve demonstrated its applicability for TAVI CT planning and are currently working on a more complex use case: risk assessment of pacemaker implantation after TAVI.

One of the key strengths of federated learning is that it offers a scalable solution to a major challenge in clinical AI—accessing diverse, high-quality data without violating privacy regulations. Given the complexity of cross-border data sharing due to differing legal frameworks, this approach presents a promising model for international collaboration.

Moreover, federated learning allows us to validate algorithms in a truly multicenter setting. This not only enhances the robustness of our models but also ensures they are better suited to real-world clinical variability. The success and planned expansion of FLOTO beyond the DZHK demonstrate the growing potential of this paradigm in advancing trustworthy AI in cardiovascular medicine.

## In your view, what are the most promising applications of AI in cardiovascular care today and what barriers still need to be overcome for widespread clinical adoption?

In my view, one of the most promising applications of AI in cardiovascular care lies in prevention and in empowering individuals to proactively monitor their cardiovascular health. The ability to extract complex diagnostic insights—as well as short- and long-term risk predictions—from low-cost, widely available signals such as ECG has the potential to fundamentally disrupt traditional diagnostic pathways. This could make early detection more accessible and cost-effective across healthcare systems.

Furthermore, I believe this shift will have profound implications for how hospitals are organized. I also expect that the core responsibilities of medical specialties will increasingly focus on treatment—ideally with more effective management of chronic conditions before they progress to severe stages.

That said, several challenges remain. These include regulatory frameworks—especially within the EU—and the need to build trust in AI systems. Fortunately, the first randomized trials are already showing very promising performance of certified AI systems in medicine. We certainly need more of such examples in cardiovascular medicine.

## As Chair of the ESC Digital & AI Summit 2025, what are the key themes or innovations you hope to highlight this year and how do they reflect the evolving role of AI in cardiology?

I’m deeply honoured to serve as Chair of the ESC Digital & AI Summit 2025. It’s both an exciting and humbling experience—especially as it’s my first time leading an event of this scale as Program Chair. I’m fortunate to be surrounded by incredibly supportive colleagues, including Folkert Asselbergs, yourself, and representatives from the ESC Digital Cardiology and AI (DCAI) group. Their collaboration makes this journey not only rewarding but also truly inspiring.

One of my main goals for this year’s Summit is to bring together diverse voices from across disciplines. In particular, I hope we attract researchers from the field of medical computer science to foster meaningful exchange with world-class cardiologists, cardiac surgeons, policymakers, and industry partners. It’s this kind of interdisciplinary dialogue that really drives innovation.

AI is now influencing nearly every area of cardiology—from imaging and diagnostics to hospital organization and workflow optimization. The Summit will reflect this breadth, with sessions covering some of the most impactful and emerging solutions, including foundation models, generative AI, multimodal approaches, federated learning, large language models, and advanced modeling techniques.

One session I’m particularly excited about is an interactive discussion on the variability of data interpretation and the limitations of current foundation models. It’s a crucial step toward improving model reliability and clinical relevance. We’re also planning live demonstrations of research prototypes, giving scientific groups the chance to engage directly with the technology and provide feedback—something I believe will spark valuable collaboration between technical experts and clinicians.

And finally, I’m especially looking forward to the keynote debate: ‘AI in cardiology – a force for progress or a path to big tech control, dehumanized medicine, and the obsolescence of physicians?’ It’s a timely and necessary conversation that reflects broader concerns about the future of medicine in the age of AI.

Overall, the Summit aims to highlight not just technological innovation, but also the ethical, practical, and human dimensions of integrating AI into cardiovascular care.

## Funding

No funding.

